# Essential Tremor in the Elderly and Risk for Dementia

**DOI:** 10.1155/2014/328765

**Published:** 2014-04-09

**Authors:** Holly A. Shill, Joseph G. Hentz, Sandra A. Jacobson, Christine Belden, Marwan N. Sabbagh, Thomas G. Beach, Erika Driver-Dunckley, Charles H. Adler

**Affiliations:** ^1^Banner Sun Health Research Institute, 10515 West Santa Fe. Drive, Sun City, AZ 85351, USA; ^2^Mayo Clinic Arizona, 13400 East Shea Boulevard, Scottsdale, AZ 85259, USA

## Abstract

The objective is to examine the risk of dementia in subjects with essential tremor (ET) involved in the Arizona Study of Aging and Neurodegenerative Disorders. All subjects were free of a neurodegenerative diagnosis at baseline and had annual motor, general neurological, and neuropsychological assessments. Subjects with ET were compared with controls for the risk of dementia. There were 83 subjects with ET and 424 subjects without tremor. Mean age at study entry was 80 ± 5.9 for ET and 76 ± 8.5 for controls. Median tremor duration was 5.2 years at study entry. Followup was a median of 5.4 years (range 0.9 to 12.1). The hazard ratio for the association between ET and dementia was 0.79 (95% CI 0.33 to 1.85). The hazard ratio for the association between tremor onset at age 65 or over, versus onset before age 65, was 2.1 (95% CI 0.24 to 18) and the hazard ratio for the association between tremor duration greater than 5 years, versus less than 5 years, was 0.46 (95% CI 0.08 to 2.6). We conclude that all elderly ET was not associated with an increased risk of dementia but that a subset of subjects with older age onset/shorter duration tremor may be at higher risk.

## 1. Introduction

Essential tremor (ET) is a common neurological condition which increases with the age of the population and contributes to significant disability in most affected patients. Previous cross-sectional studies have shown that patients with ET may have minor cognitive deficits on formal testing although most of these older studies have been done with advanced ET patients being assessed for deep brain stimulation as treatment for tremor [1, 2]. More recently, a population study of ET demonstrated that more mildly affected, largely untreated ET individuals may be more likely to complain of memory problems and have deficits at testing [3]. This same population was more likely to have prevalent dementia, largely driven by elderly onset ET [4]. In those nondemented at baseline, incident dementia was greater in ET [5].

This study seeks to compare the risk of developing dementia in subjects with ET versus controls without tremor in a large, well-categorized cohort of individuals involved in a longitudinal aging study, the Arizona Study of Aging and Neurodegenerative Disorders (AZSAND).

## 2. Methods

Participants without dementia or another neurodegenerative disorder at study entry and who had at least one follow-up visit were selected from the 23 May 2013 version of the AZSAND database which included 3300 subjects. All participants, both cases and controls, were initially recruited into the study largely as a result of lectures and community awareness within the catchment area of Maricopa County, Arizona, and provided informed consent approved by Banner IRB. Beginning in 1997, all participants had an annual UPDRS and Fahn Tolosa Marin (FTM) tremor scale [6] done by a movement disorder neurologist (Holly A. Shill, Charles H. Adler, Erika Driver-Dunckley) who annually classified the subject movement diagnosis based on their current examination and review of medical records. Participants were diagnosed with ET if they had carried a clinical diagnosis of ET and the examination was consistent with that diagnosis or if they had an isolated head or voice tremor without dystonia. If the participants did not have a diagnosis of ET but did have a postural or kinetic hand tremor score of ≥2 on the FTM scale without secondary cause, then they were given a research diagnosis of ET. If the participants had a postural or terminal tremor of the hands <2, they were categorized as having tremor NOS. These participants were then reclassified on subsequent annual examinations as ET if they had persistent tremor greater than 3 years without secondary cause. All had annual neuropsychological testing which included at a minimum: WAIS-III Digit Span [7], auditory verbal learning test (Rey AVLT) [8], controlled oral word association (COWAT) [9], category fluency, Boston naming test (BNT) [10], Clock Drawing [10], Judgment of Line Orientation (JLO) [9], Trails Part A/B [11], STROOP [12], and MMSE [13]. General medical and neurological examinations were performed annually. All clinical information was used to categorize cognitive status at consensus conference staffed by a behavioral neurologist, psychiatrist, and neuropsychologist. Dementia was defined according to DSM-IV criteria. A subset of the cohort had Apo E genotyping done.

Baseline characteristics of both groups were compared using *t*-test or chi square as appropriate. The incidence of dementia in participants with ET was compared to that of controls without action tremor by using Cox regression. The incidence of dementia in ET with tremor onset at age 65 years or greater was compared to that of ET with tremor onset before the age of 65 by using Cox regression as previous reviews [14] have suggested subtyping ET based on beginning prior to or after the age of 65. The incidence of dementia in those with tremor duration greater than the median was compared to those with tremor less than the median. The incidence of dementia was compared in participants with ET compared to controls after excluding those with MCI at baseline.

## 3. Results

Out of 3300 individuals in the database, 1266 had baseline cognitive and motor testing and 1052 were not demented at baseline. The majority of those who did not have both standardized assessments were enrolled in the program prior to 1997. ET was present in 141 participants, 679 did not have tremor, and 232 had tremor that did not meet criteria to be included (low amplitude, nonpersisting tremor, or secondary tremor). After excluding participants with another baseline neurodegenerative disorder such as parkinsonism (30 in the tremor group and 160 in the nontremor group) and including only those having at least two movement and cognitive exams, the final analysis included 83 participants with tremor and 424 controls.

The proportion of women was lower in the tremor group than the control group (Table 1). Mean age was higher in the tremor group at baseline (80 ± 5.9 versus 76.9 ± 8.5, *P* = 0.002). Age at tremor onset ranged from near birth to 91 years (mean 66 ± 21), and the duration of tremor at study entry ranged from 0 to 72 years (mean 14 ± 19, median 5.2 years). The proportion of ApoE *ε*4 carriers was the same in both groups (13/55, 23.6% tremor versus 57/238, 23.9% controls). Followup ranged from 0.9 to 12.1 years, with a median of 5.4 years.

**Figure 1:**
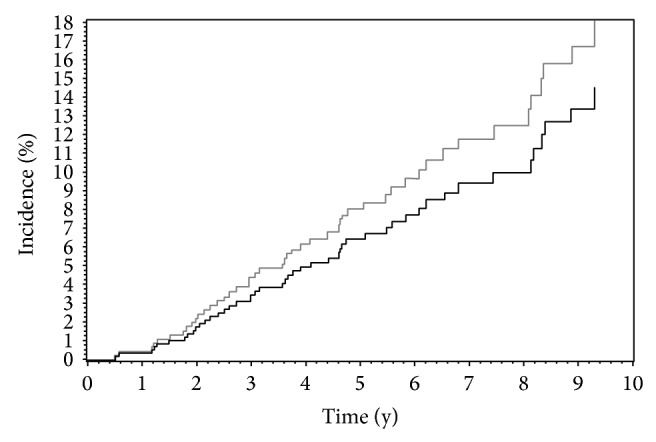
Incidence of dementia after study entry in 83 subjects with ET (black) and 424 subjects without ET (gray).

Baseline neurocognitive status was similar between both groups (Table 1). The incidence of dementia was not different in the tremor group compared to the control group (Figure 1). The incidence of dementia within 5 years of study entry was 6% for ET and 8% for controls (95% CI 1% to 11% for tremor, and 5% to 11% for controls). The hazard ratio for the association between ET and dementia was 0.79 (95% CI 0.33 to 1.85; *P* = 0.58). Adjustment for age and sex, or for age, sex, and ApoE *ε*4, nonsignificantly decreased the hazard ratio for the association between tremor and dementia (HR 0.50, 95% CI 0.21 to 1.20, *P* = 0.12; or HR 0.46, 95% CI 0.17 to 1.23, *P* = 0.12). Excluding participants with MCI at baseline, the hazard ratio for the development of dementia was 1.06 (95% CI 0.17–1.23). The sample was too small to assess the relationship between dementia and age of tremor onset or duration of tremor. The hazard ratio for the association between onset at age 65 or over, versus onset before age 65, was 2.1 (95% CI 0.24 to 18). The hazard ratio for the association between tremor duration greater than 5 years, versus tremor duration no more than 5 years, was 0.46 (95% CI 0.08 to 2.6).

## 4. Discussion

The main finding of this study was that all subjects with ET did not develop dementia at a higher rate than control subjects without tremor in this well-categorized longitudinal study. These results are in contrast to the findings in the Spanish population study reported in 2007 [5] where they reported an unadjusted relative risk of dementia of 2.08 (95% CI = 1.24–3.50) in ET. Our study found a relative risk of 0.79 (95% CI 0.33 to 1.85). While the Spanish population study enrolled over 3000 subjects, the older age at baseline for our group increases the relative percentages of tremor and dementia making the absolute numbers fairly comparable. Advantages of the current study are that all subjects were followed annually with a more comprehensive standardized motor and neuropsychological test battery, were assessed in the clinic regardless of entry diagnosis, and had longer duration median followup. Further, both the tremor and control groups were more similarly matched in terms of level of education compared with the previous study (although this was corrected for in the final analysis).

In an aging study in New York [15] investigators found a higher amount of dementia in their cross-sectional sample but found a nonsignificant adjusted incident dementia risk of 1.64 for dementia in prospectively followed cases. This is similar to our findings with advantages of the current study being prospective, in person assessments of all participants (tremor and controls) by movement disorder and behavioral neurology specialists, similar baseline characteristics of both patient populations, and longer duration followup.

Previous study has suggested that older age of onset of tremor might be a higher risk for cognitive decline [4, 5]. We did not have a large enough number of each group to specifically examine this with statistical power (the hazard ratio for the association between onset at age 65 or over, versus onset before age 65, was 2.1 but with very large confidence intervals of 0.24 to 18). However, taken together with the other finding of increased dementia in shorter duration tremor, this suggests that older onset tremor might be at higher risk although more subjects with very long duration tremor are needed to say this with confidence. It is worth considering that the age where this risk goes up might be high as we did not find an increased risk for the group as a whole with a mean age of 80 at study entry and a median tremor duration of 5.2 years.

Alternately, dementia in tremor patients develops relatively rapidly after the onset of tremor which would have resulted in exclusion of these subjects from our study (left censorship). If true, this might suggest that some action tremor could be an early biomarker of subsequent cognitive decline.

One limitation of our study is that our population may not be representative of the entire ET population. However, subjects with ET were not typically recruited into our study based on presence of tremor, but rather they were mostly entered in the study as elderly controls for the purposes of comparison to cases of AD and PD and therefore were recruited from a very similar demographic pool as controls [16]. As a result, the comparisons between the two groups based on presence or absence of tremor, rather than a formal medical diagnosis of ET, are likely valid for this type of study. The risk of dementia in this entire cohort is similar to other population studies with similar demographics [17].

In summary, we did not find a link between tremor and dementia in the overall group. However, there was suggestion that elderly onset, shorter duration tremor might be at higher risk. While some have proposed separating ET into categories based on age of onset of tremor [14], it is still not clear yet if there are meaningful clinical differences between these groups with respect to risk for cognitive decline or parkinsonism [18].

## Figures and Tables

**Table 1:** 

	ET	Control	*P*
Age (y); mean (SD), *N*	80.0 (5.9), 83	76.8 (8.5), 424	0.002
Women	36/83 (43%)	30/424 (71%)	<0.001
Education (y); mean (SD), *N*	15.5 (2.8), 83	15.0 (2.7), 424	0.07
Education >12 y	64/83 (77%)	314/424 (74%)	0.56
ApoE *ε*4 carrier	13/56 (23%)	58/239 (24%)	0.87
Followup (y); mean (SD), *N*	5.5 (2.7), 83	5.8 (2.9), 424	0.30
Baseline MCI	6/83 (7%)	39/424 (9%)	0.56
MMSE (0–30); mean (SD), *N*	28.7 (1.6), 82	28.8 (1.6), 417	0.61
JLO (0–30); mean (SD), *N*	23.7 (4.1), 73	23.5 (4.1), 354	0.73
Clock drawing (0–10); mean (SD), *N*	9.32 (0.99), 80	9.30 (1.02), 421	0.87
Stroop interference (0–150); mean (SD), *N*	31.0 (9.0), 81	32.1 (9.3), 408	0.34
COWA total (0–120); mean (SD), *N*	37 (11), 82	38 (12), 421	0.71
Animal fluency; mean (SD), *N*	16.6 (4.3), 82	17.2 (4.6), 422	0.28
AVLT total learning (0–75); mean (SD), *N*	43.3 (9.3), 82	45.0 (10.3), 422	0.17
AVLT long-term memory A7 (0–15); mean (SD), *N*	8.7 (3.7), 82	9.0 (3.3), 423	0.54
AVLT percent recall (%); mean (SD), *N*	76 (24), 82	78 (21), 423	0.61
Digit span forward correct (0–12); mean (SD), *N*	7.7 (2.1), 13	7.8 (1.7), 53	0.86
Digit span backward correct (0–12); mean (SD), *N*	6.7 (2.3), 13	6.3 (2.1), 53	0.60
BNT (0–30); mean (SD), *N*	27.4 (1.9), 13	26.7 (2.4), 52	0.37
TMT-A (0–150 seconds); mean (SD), *N*	43 (17), 81	38 (17), 419	0.02
TMT-B (0–300 seconds); mean (SD), *N*	109 (47), 81	99 (47), 417	0.08
